# Total knee arthroplasty with the least-constrained implant possible for type II valgus knee > 20°: a 3–14 years’ follow-up

**DOI:** 10.1186/s42836-020-00036-6

**Published:** 2020-06-23

**Authors:** Jiangdong Ren, Xiaogang Zhang, Wuhuzi Wulamu, Nuerailijiang Yushan, Abudousaimi Aaimaiti, Li Cao

**Affiliations:** 1st affiliated Hospital of Xinjiang Medical University, Ürümqi, China

**Keywords:** Total knee arthroplasty, Valgus, Lateral release

## Abstract

**Objective:**

To estimate the midterm outcome of primary total knee arthroplasty for severe valgus deformity using selective release of tight lateral structures and the least-constrained implant.

**Methods:**

We performed total knee arthroplasty on 65 consecutive type II knees with valgus deformity> 20°. Surgery was done via a medial parapatellar approach. Conventional bone cutting was done with selective lateral soft tissue release, and the least-constrained total knee prosthesis possible was used. Posterior stabilized implants were employed in most knees, except for three knees that required the implantation of constrained condylar knee prostheses. The average duration of follow-up lasted for 10.5 years.

**Results:**

Preoperatively, average valgus was 30.6°, and average range of motion was 43.7° (range, 0–80°). Postoperatively, average valgus was 7.3° and average range of motion was 110.6° (range, 80–130°). The lateral collateral ligament and iliotibial band were released in all knees, and release of the popliteus tendon was required in two knees. Stable flexion and extension gaps were achieved in most cases, except for two that had medial side instability. Follow-up showed that stability was maintained.

**Conclusions:**

This surgical technique combined selective lateral soft tissue release with use of the least-constrained implant possible and was effective for severe valgus deformities of the knee, with good clinical results.

## Introduction

Approximately 10% of patients requiring total knee arthroplasty (TKA) have valgus deformity [1]. This valgus deformity may be caused by osteoarthritis, rheumatoid arthritis, post-traumatic arthritis, or metabolic bone disease. The valgus knee has been classified into three types: type I has minimal valgus without medial soft tissue stretching; type II has a more substantial deformity (> 10°) with medial soft tissue stretching, and type III refers to a severe osseous deformity after prior osteotomy with an incompetent medial soft tissue sleeve [2]. Primary TKA for valgus deformity is a formidable surgical challenge, especially for severe valgus knees type II and III (> 20° valgus from the anatomic normal of 5° valgus) [3].

The valgus deformity consists of two components. The first component is osseous deficiency of the lateral femoral condyle and/or tibial plateau, distal femoral hypoplasia, posterior femoral condylar erosion, external rotation deformity of the distal femur, secondary remodeling of the femoral and tibial metadiaphyseal region, and patellar maltracking. The second is soft tissue contracture due to tight lateral structures, such as the iliotibial band (ITB), lateral collateral ligament (LCL), popliteus tendon, posterolateral capsule, and hamstring muscles [4]. It is crucial to correct the deformities and establish ligament balance to ensure a good, durable and functional result in TKA. However, achieving these two objectives may be difficult in the cases of severe fixed valgus deformity [2, 5], especially in patients suffering from knee deformity > 20°. After restoring bony alignment and positioning of the articular surfaces at the time of surgery, a strategy is necessary to ensure correct soft tissue balance throughout the range of motion.

An ideal TKA should provide both immediate and long-term stability and cause no notable increase in component loosening or wear rates. Despite advances in instrumentation for bone resection and alignment, correcting valgus deformity without relying on the use of a constrained implant remains a challenge for many surgeons [6]. Several authors have raised concerns that high constraint [constrained condylar knee (CCK)] prostheses and hinge prostheses are associated with a higher risk of loosening and exposure to technical difficulties in the case of revision [7]. Systematic recourse to implant high-level constraint prostheses is questionable, and several authors have proposed adjustment of this indication to the ligament balancing problems [8].

With severe valgus knee, the contracted lateral soft tissue structures (the ITB, LCL, popliteus tendon, and posterolateral capsule) should be released step-by-step on the lateral side. Eventual attenuation of the corresponding medial structures occurs in extreme cases. Although numerous techniques have been proposed for the establishment of balanced soft tissue tension, there is currently no consensus regarding the sequence in which one or all of these structures should be addressed [2–6]. Furthermore, although cadaveric studies have previously assessed the functional role of the lateral soft tissue structures, * in vivo* data on the effectiveness of these techniques have been scanty [9].

In this study, we performed TKA on severe valgus knees via a medial parapatellar approach. Conventional bone cutting was done with selective release of lateral soft tissues, and the least-constrained total knee prosthesis possible was used. The purpose of this novel method was to avoid late-onset instability and the need for a constrained implant. We selectively released the tight lateral structures in a step-by-step fashion (by palpating and releasing tight areas), and the lateral retinaculum in the cases of *with* patellar maltracking. We hypothesized that this method would: (1) attain and maintain correction of the frontal plane deformity; (2) restore patellar tracking and well restore the patellar function; and (3) avoid complications associated with extensive release and the need for a constrained implant.

## Materials and methods

### Patients and follow-up evaluation

We retrospectively analyzed the records of 62 patients with 65 severe type II valgus knee deformities who underwent TKA between January 2004 and December 2015. Three patients had bilateral knee valgus deformity plus hypoplasia of the lateral condyle of the femur. No patients had preoperative fixed flexion contracture. There were 15 males and 47 females, with an average age of 65 years (ranging from 41 to 78 years). There were 49 cases of osteoarthritis, 13 cases of rheumatoid arthritis, and 7 cases complicated with patella dislocation, including 2 rheumatoid and 5 osteoarthritis. We performed standard osteotomy via the medial parapatellar approach, conducted selective lateral soft tissue release, and used posterior stabilized (PS) implants in all cases except those in which a high level of constraint was required. Two patients (two knees) died, and two patients (two knees) were lost to follow-up, leaving 58 patients (61 knees) who were followed up for 10.5 years on average (range, 3–14 years). Fifty-one patients were clinically evaluated in person by the authors, and 11 were interviewed by telephone. Patients were assessed against the Knee Society Knee and Functional Scoring System [10], and the tibiofemoral angle (angle between the femur and the tibia anatomic axis) was radiologically measured on a weight-bearing anteroposterior (AP) radiograph of the knee.

### Surgical technique

All surgeries were performed by the same senior surgeon, with the patient under epidural or general anesthesia with a tourniquet. A cemented, non-constrained fixed-bearing, PS knee prosthesis of the same design (NexGen Legacy Posterior Stabilized Prosthesis, Zimmer, Warsaw, Ind) was used in each case, except for three cases in whom the constrained condylar knee (CCK) prosthesis (LCCK Zimmer, Warsaw, Ind) was used.

**Fig. 1:**
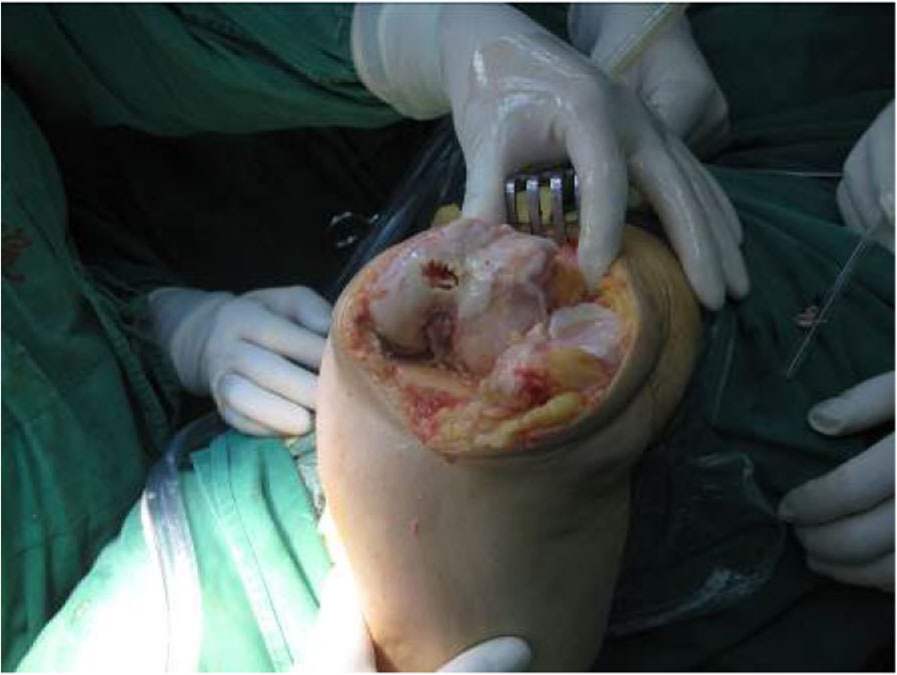
The entry point of the intramedullary rod was medialized by 1–2 mm

**Fig. 2:**
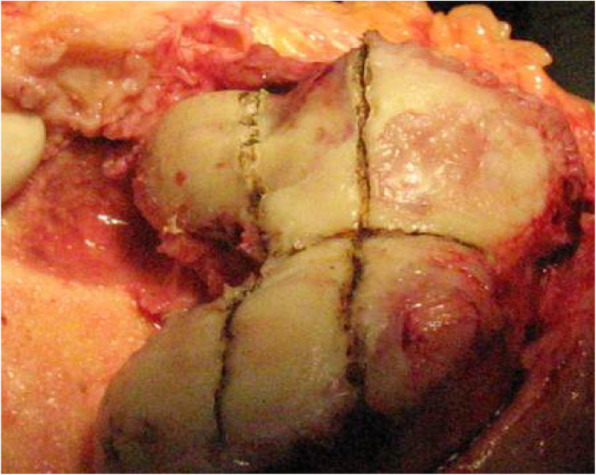
The epicondylar axis and the Whiteside line were also determined and used as a control for bone resection

Intraoperatively, the same method was used in all cases: surgery was carried out through a midline skin incision and medial parapatellar arthrotomy, and the medial soft tissues were not released from the proximal part of the tibia. After exposure of the knee joint, any osteophytes were removed. The patellofemoral ligament was released to allow eversion and lateral dislocation of the patella, and the knee was fully flexed to expose the cruciate ligaments and menisci for excision. Basic tibial bone resection was then performed in the standard manner. An extramedullary tibial guide was used in each case, and the tibial resection surface was perpendicular to the tibial mechanical axis. An intramedullary guide was used for femoral resection in all knees. Because of the osseous deficiency of the lateral femoral condyle, the entry point of the intramedullary rod was medialized by 1–2 mm (Fig. 1). Distal femoral resection of 10 mm was performed with the knee in 3–4° of valgus in relation to the anatomical axis in order to protect against undercorrection of the underlying deformity [2]. Autologous bone grafting was done if there was bone deficiency of the distal lateral femoral condyle by impacting a small graft or using K-wire for large graft. The femoral component rotation was then determined at 90° of flexion using a tensor with separated distraction of both components. The epicondylar axis and the Whiteside line were also determined and used as a control (Fig. 2). The posterior cutting was performed parallel to the tibia.

The symmetry of the extension space was assessed after laminar spreaders were inserted into the medial and lateral femorotibial space with the knee in extension and flexion. In all cases, the lateral soft tissue structures contracted and required release. We found that the knees that were too tight laterally in flexion were mostly tight in extension. For severe valgus deformity, especially when > 20°, it was inadequate to use the pie crust technique alone to release the posterolateral capsule.

**Fig. 3:**
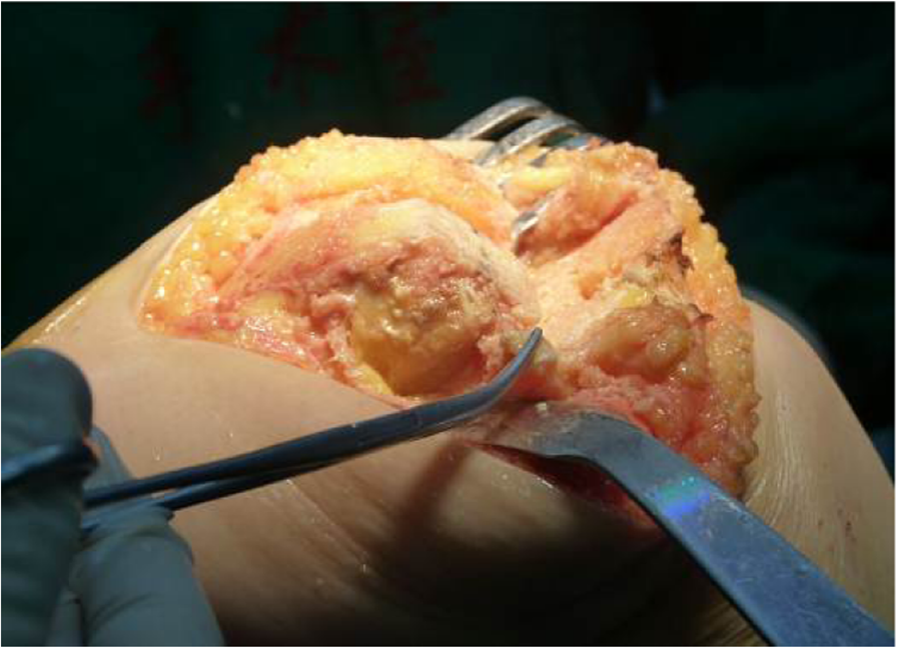
The popliteus tendon attachment to the femur was released when the lateral tightness correlated with internal rotational contracture

**Fig. 4:**
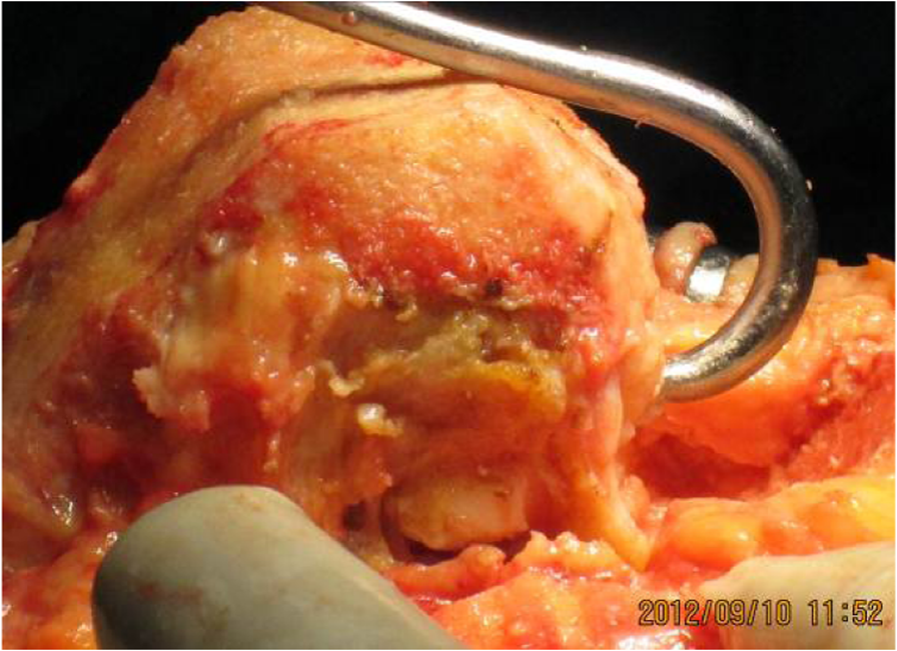
The LCL was detached from the lateral femoral condyle if necessary

For knees that were tight laterally in both flexion and extension, the flexion was balanced firstly. Any osteophytes and the hyperplastic sesamoid bones were then removed at the posterolateral corner of the knee in flexion. The tightness was re-tested on the lateral side of the knee, and the LCL was detached from the lateral femoral condyle, if necessary (Fig. 3). Additional release of the popliteus tendon was done as necessary to achieve ligament balance. The popliteus tendon attachment to the femur was also released when the lateral tightness correlated with internal rotational contracture (Fig. 4). Then, the ITB was released in the extended position using the pie crust technique or, in some cases, was cut transversely.

In knees that were mostly tight laterally in extension, the LCL and the popliteus tendon were left intact, and the ITB was released using the pie crust technique or cut transversely in some cases. If the lateral side was still a little bit tight, the LCL was pie crusted from the attachment of the femur using a 30 G needle.

An appropriately-sized spacer block that filled the flexion space was then inserted, and the balance of the medial and lateral gaps was evaluated. If inadequate balance was present, the aforementioned steps were repeated until medial and lateral soft tension was symmetric in flexion and extension.

**Fig. 5:**
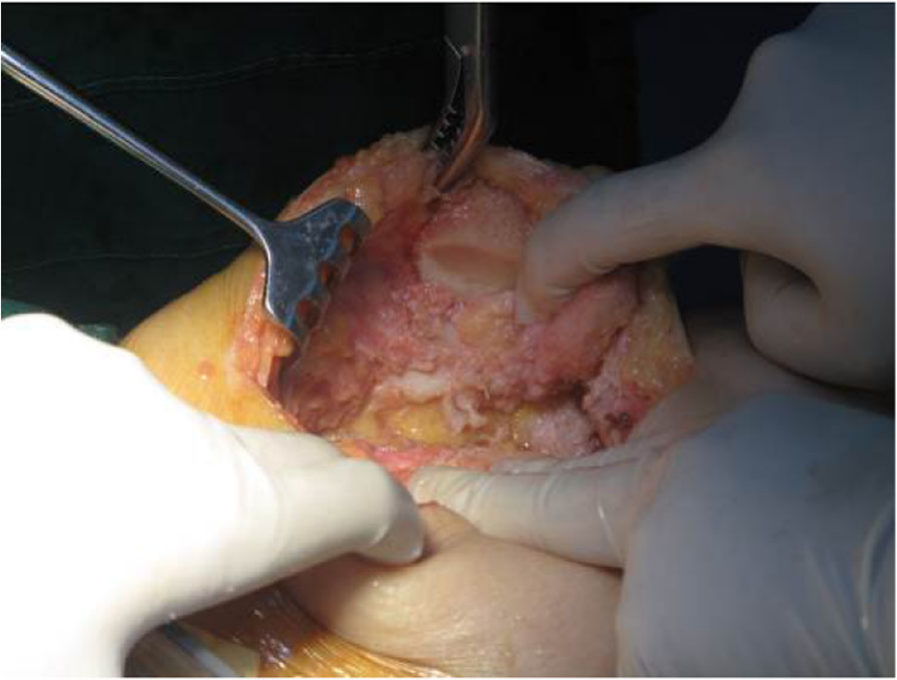
If lateral patellar tilt or maltracking was present, the lateral patellar retinaculum was released

Patellar tracking was checked with the trial components implanted using the no-thumb technique and with traction applied to the quadriceps tendon by a clamp. If lateral patellar tilt or maltracking was present, the lateral patellar retinaculum was released. A scalpel was used to make a vertical cut in the retinaculum just below the vessels to reveal the adjacent subcutaneous fatty layer (Fig. 5). For complete patellar dislocations, the extensor mechanism was tight after patellar reduction, and the quadriceps tendon was released using the pie crust technique.

The indications for patellar resurfacing were severe patellar deformity and maltracking after lateral retinaculum release. The patella was re-surfaced with a polyethylene dome in five cases. The real components were implanted once the knee was well-balanced.

## Results

### Soft tissue release

All patients required selective lateral soft tissue release, including release of the ITB, LCL, partial or complete. No case required release of the posterolateral capsule or the lateral gastrocnemius from the femur, while two of the 62 patients required popliteus tendon release from the femur.

### Clinical and radiographic results

**Fig. 6:**
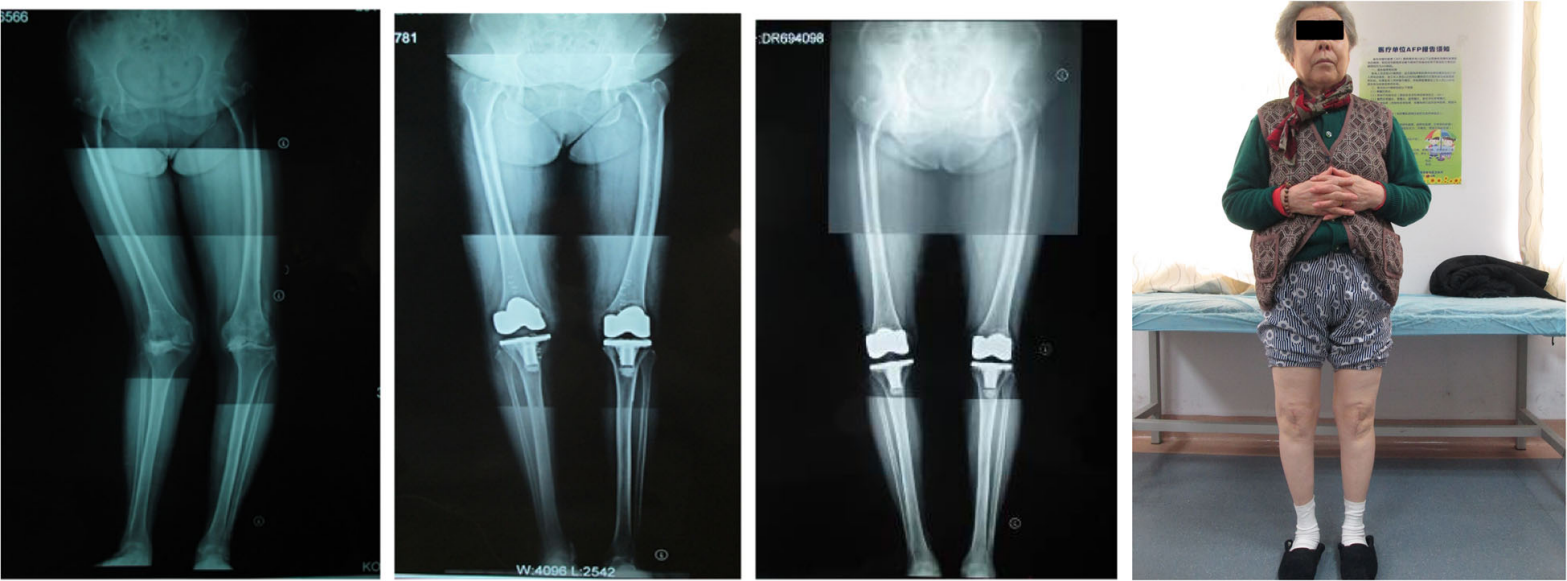
A seventy-three years old female with right severe knee valgus deformity, 7 years after surgery. Conponents were well-fixed without loosening, and no radiolucent line was observed

The 58 included patients (involving 61 knees and including three cases of bilateral TKA) were followed up for an average time of 10.5 years (range: 3–14 years). Clinical results were all good or excellent, with a mean Knee Society score of 95 (range: 85–100). The mean Knee Society functional score was 73 (range, 45–100). Three patients had scores less than 65, and they were limited by other comorbidities, including spinal stenosis, neuropathy, and chronic obstructive pulmonary disease. Mean preoperative range of motion was 43.7° (range, 0–80°), and was improved postoperatively to a mean of 110.6° (range: 80–130°). The mean preoperative femorotibial anatomic alignment measured on a weight-bearing AP radiograph was 30.6° valgus (range: 21–45° valgus). Postoperative weight-bearing AP radiographs of the knee confirmed that the mean femorotibial alignment at the last follow-up was 7.3° valgus (range, 0–10° valgus) (Fig. 6). At the last follow-up, no patient complained of instability, none of the patients examined had any medial or lateral laxity, and there were no cases of radiographic loosening, medial or lateral condylar liftoff, or gross polyethylene wear.

### Complications

One patient had patellar subluxation caused by a fall that occurred when the knee was in 60° flexion, but the patient was satisfied with the function and refused further treatment. Two cases of medial instability at the 6-month follow-up were treated by using articular braces. Four cases of transient palsy of the peroneal nerve recovered within 6 months. There were no such complications as infection, fatal pulmonary embolism, patellar dislocation, knee instability or component loosening.

## Discussion

### The surgical approach in TKA for the valgus knee

Severe valgus knee deformity, especially when valgus angle > 20°, is thought to be the biggest challenge posed by TKA, and numerous alternatives have been advocated to create balanced extension and flexion gaps and restore the mechanical alignment of the lower limb [1, 3, 4]. However, there is no consensus regarding the most appropriate surgical approach, technique of soft tissue balancing, and components for arthritic valgus knees [11–13]. Several studies compared the clinical results of the medial *versus *the lateral approach in TKA for the valgus knee, but results have been conflicting .

The lateral approach for valgus TKA is widely accepted and provides good surgical and long-term functional outcomes. The potential disadvantages of this approach include difficulty in patellar eversion, which sometimes requires tibial tubercle osteotomy, with which many surgeons are less familiar [14]. A recent study comparing the short-term results of two surgical approaches in TKA for patients with valgus deformity suggested that the lateral approach can directly adjust the tight structures, is less invasive to the quadriceps muscle, causes less vascular compromise to the patella, and provides a better postoperative range of motion [15]. However, the valgus angle was < 20° in this study, and the theoretical advantages of the lateral approach were not clinically verified for severe valgus knee deformity. The medial parapatellar approach is generally regarded as the standard TKA approach in the valgus knee, since it provides a good surgical and long-term functional outcome [11]. In the present study, 62 patients with severe valgus knee deformity received TKA through the medial parapatellar approach, standard osteotomy and selective lateral soft tissue release and all cases showed improvement in gap balance, joint stability, and alignment correction.

### Correction of the frontal plane deformity

With valgus knee deformity, bone resection should be carried out differently in order to correct the deformities. Surgeons should pay attention to valgus knee bony hypoplastic variables, because these characteristics can influence limb alignment, component rotation and patellar tracking [16]. This helped to attenuate or stretch the medial collateral ligament to prevent future contracture. During femoral component rotation, the surgeon had to pay heed to lateral condylar hypoplasia that could result in conspicuous internal rotation of the components if a posterior reference was used.

Achieving adequate soft tissue balance in valgus knees during TKA remains a challenge to arthroplasty surgeons, and a favorable surgical outcome depends on multiple factors, including postoperative lower limb alignment, joint stability, patellar tracking and the range of motion of the knee joint [17, 18]. It is generally accepted that lateral structural release is necessary with knees suffering from severe valgus [1, 16, 19]. Nonetheless, the best sequence of release and the best technique to achieve these releases is still controversial.

Numerous alternative soft tissue balancing procedures have been advocated over the last 20 years [2, 20]. Insall *et al*. were the first to report the pie crust soft tissue balancing method and described the ‘inside-out’ or ‘outside-inside’ technique through the taut posterior cruciate ligament or ITB with the knee in full extension [21, 22]. Ranawat recommended transverse division of the posterolateral capsule above the joint line [1, 2]. Whiteside suggested the sequential release of the ITB, popliteus, LCL and the lateral head of gastrocnemius, and tibial tubercle transfer when the Q angle was > 20° [20]. Other suggestions include a sequential three-step lateral release strategy [19], medial soft tissue advancement combined with lateral release [23], and a lateral capsular approach [14, 15]. However, in the present case series, the release of tight lateral soft tissue using the pie crust technique and transverse division of the posterolateral capsule was insufficient.

Most severe valgus knees, especially knees with fixed deformity > 20°, have complex bony deformities, including distal femoral hypoplasia, osseous deficiency of the lateral femoral condyle and/or tibial plateaus, posterior femoral condylar erosion, external rotation deformity of the distal femur, and patellar maltracking. These cases also have tightly contracted lateral structures, such as the ITB, LCL, popliteus tendon, posterolateral capsule, and hamstring muscles. Release of ligaments according to an arbitrary protocol that does not consider ligament function in flexion and extension could result in complex instability problems. We believe selective lateral soft tissue release (ITB, LCL, popliteus tendon) and maintenance of the integrity of other contractured soft tissue via the medial parapatellar approach, with severe deformities, are appropriate and necessary for long-term implant survival. Sixty-two of the 65 knees underwent selective lateral soft tissue release. In most cases, the lateral soft tissue was tight in both flexion and extension. The ITB was released in the extended position using the pie crust technique (or was cut transversely in some cases), which then allowed us to remove the osteophytes and hyperplastic sesamoid bones. In three cases, the flexion gap was balanced when any osteophytes and the hypertrophic sesamoid bones were removed at the posterolateral corner in flexion. For knees that were still tight laterally in both flexion and extension, the tightness was palpated with the surgeon’s finger, and then the LCL was detached gradually by electrocautery from the lateral femoral condyle until a rectangular flexion and extension gap was achieved (Fig. 4). Care was exercised to maintain integrity of the lateral capsule and soft tissues. The LCL was completely detached in 20 cases, while partial detachment was sufficient in others. Two additional releases of the popliteus tendon were done as necessary to achieve ligament balance. In this situation, the other lateral soft tissue structures must be left intact.

### Patellar tracking and function

For type II knees with valgus > 20° plus concurrent patellar dislocation, we imbricated the medial capsule and vastus medialis muscle to avoid patellar maltracking and strengthen the attenuated medial collateral ligament, which rendered it convenient to perform selective inside-out lateral soft tissue release.

The postoperative clinical scores were all good or excellent. The postoperative functional scores were generally good or excellent, except for three patients who were limited by other medical comorbidities, namely spinal stenosis, neuropathy, and chronic obstructive pulmonary disease. There were no cases, at the last follow-up, of patellar instability or knee instability, indicating that the outcomes regarding patellar tracking and function were good.

### Complications and implant suitability

We found that in cases with fixed valgus > 20° plus concurrent patellar dislocation, it was difficult to suture the lateral incision. Hence, using the medial parapatellar approach decreased the risk of infection and wound complications. Only two patients had postoperative instability. They had medial instability that was treated by using articular braces. Final follow-up revealed no postoperative medial or lateral laxity, radiographic loosening, medial or lateral condylar liftoff or gross polyethylene wear.

With TKA for severe valgus knee deformity, selecting right implant is critical for achieving a long-term postoperative survival rate of the prosthesis. In general, implant should be selected on the basis of the preoperative radiological and clinical evaluation, and the final decision should be made after the bone cutting and knee soft tissue balancing. In the present group of type II patients, three cases of 30° valgus deformity were complicated with patellar dislocation. After the bone cuts, soft tissue cuts, radiological and clinical evaluation, a 4-mm instability remained even after imbrication of the medial collateral ligament. Hence, we had to use the CCK prosthesis. Two cases of medial instability < 4 mm were treated by using articular braces for 6 months.

Some surgeons believe that hinged implants are suitable for elderly patients with severe irreducible valgus, as the severe valgus deformity may require more aggressive release [21]. However, others believe that these high-constraint prostheses and hinge prostheses have a higher risk of loosening and exposure to technical difficulties in the case of revision. Some studies achieved good results using non-constrained prostheses in patients with moderate to severe valgus deformity [20]. Others reported good results using posterior cruciate ligament-retaining designs with various soft tissue balancing techniques [2, 16], and suggest that cruciate-retaining implants could be used in a wide range of patients with valgus osteoarthritis [24]. A 15-year follow-up study showed that posterior cruciate ligament-retaining prostheses significantly improved the survival rate in comparison with posterior cruciate ligament-stabilizing prostheses [25].

We believe that there are potential advantages to using a PS design in the correction of severe valgus deformity. Under severe valgus conditions, this technique involves complete resection of the posterior cruciate ligament, obviating some difficulties associated with a cruciate-retaining design [2].

## Conclusions

Full correction of the valgus deformity without late-onset instability was achieved by applying our principle of releasing tension wherever it was found. The PS design was adopted and non-constrained prostheses were used in all but three cases. This technique, which combined selective lateral soft tissue release with the least-constrained implant possible, provided a safe, reliable way of addressing most severe valgus deformities of the knee and achieved good clinical results.

## Data Availability

No supporting data are in connection with this article from other departments. (Supporting data are all stored in our centre.)

## Abbreviations

TKA: Total knee arthroplasty
ITB: Iliotibial band
LCL: Lateral collateral ligament
CCK: Constrained condylar knee
PS: Posterior stabilized
AP: Anteroposterior
